# Evaluation of the nutritional status of workers of transformation industries adherent to the Brazilian Workers’ Food Program. A comparative study

**DOI:** 10.1371/journal.pone.0171821

**Published:** 2017-02-09

**Authors:** Ingrid W. Leal Bezerra, António Gouveia Oliveira, Liana G. B. Pinheiro, Célia M. M. Morais, Luciano M. B. Sampaio

**Affiliations:** 1 Department of Nutrition, Centro de Ciências da Saúde, Universidade Federal do Rio Grande do Norte, Natal, Rio Grande do Norte, Brazil; 2 Department of Pharmacy, Centro de Ciências da Saúde, Universidade Federal do Rio Grande do Norte, Natal, Rio Grande do Norte, Brazil; 3 Department of Administration Sciences, Universidade Federal do Rio Grande do Norte, Natal, Rio Grande do Norte, Brazil; Institut de recherche pour le developpement, FRANCE

## Abstract

The objective of this study was to assess whether the Brazilian Workers’ Food Program (WFP) is associated with changes in the nutritional status of workers in the transformation industry. We conducted a cross-sectional, observational, comparative study, based on prospectively collected data from a combined stratified and two-stage probability sample of workers from 26 small and medium size companies, 13 adherent and 13 non-adherent to the WFP, in the food, mining and textile sectors. Study variables were body mass index (BMI), waist circumference (WC), and dietary intake at lunch obtained by 24-hour dietary recall. Data were analyzed with nested mixed effects linear regression with adjustment by subject variables. Sampling weights were applied in computing population parameters. The final sample consisted of 1069 workers, 541 from WFP-adherent and 528 from WFP non-adherent companies. The groups were different only in education level, income and in-house training. Workers in WFP-adherent companies have greater BMI (27.0 kg/m2 vs. 26.0 kg/m2, p = 0.002) and WC (87.9 cm vs. 86.5, p = 0.04), higher prevalence of excessive weight (62.6% vs. 55.5%, p<0.001) and of increased WC (49.1% vs. 39.9%). Workers of WFP companies have lower intake of saturated fat (–1.34 g, p<0.01) and sodium (–0.3 g, p<0.01) at lunch. In conclusion, this study showed that workers of companies adherent to the Brazilian WFP have greater rates of excessive weight and increased cardiovascular risk—a negative finding—as well as lower intake of sodium and saturated fat—a positive finding. Therefore, the WFP needs to be revisited and its aims redefined according to the current epidemiological status of the target population of the program.

## Introduction

Food and nutrition are recognized as human rights in the Universal Declaration of Human Rights, in the International Covenant on Economic, Social and Cultural Rights and have entered the Brazilian legislation since 1992.[1] The State, therefore, is bound responsible for the development of actions aimed at the realization of these rights and this is made through public policies.

Faced with this challenge, Brazil has been engaged, especially in recent decades, in ensuring food and nutrition security to individuals and communities. To this end, multi- and inter-sectorial strategies have been adopted, with actions that ranged from the production to the consumption of food, that look differently to the various strata of society, that targeted specific groups, e.g, students, workers, or that allowed an interface between more than one policy in order to define responsibilities for those groups.[2]

In the case of workers, the public policy exclusive to that segment of society at the national level is the *Programa de Alimentação do Trabalhador* (Workers’ Food Program, WFP). The WFP was established by Law No. 6,321, of April 14, 1976, as a tax benefits policy to companies that offered meals to their employees. Its main objective is the improvement of the nutritional status of workers, on the assumption that well-fed individuals will have greater incentive to work, thereby generating a positive impact on the productivity of companies.[3,4] According to the Brazilian Ministry of Labor, as of December 2015 the program had 236,970 registered companies, with a coverage of 19.5 million workers.[5]

The WFP is a policy of voluntary adhesion aimed at the formal labor market, which offers to companies opting for the payment of corporate tax according to the actual profit method a tax exemption of the amount invested in workers' nutrition, up to a limit of 4% of all taxable profit in the previous year. Low-income workers (less than five minimum wages) represent the priority range of coverage, but the program does not exclude those whose income exceeds this limit. Companies affiliated to the WFP may provide meals either in-house or outsourcing the service and, in this case, both the contractor and the outsourced company must have joined the Program. The distribution of a meal ticket and/or a meal, and the distribution of basic food baskets are also acceptable outsourced modes. Regardless of the method adopted for the provision of meal, the affiliated company can offer its employees one or more meals a day, provided that the exemption limit is maintained. [5] In order to stay in the Program, companies must comply with nutritional criteria, set out in specific legislation, for the supply of food to the workers. Menus must be planned and overseen by a legally qualified dietitian, must meet the minimum daily quota of energy, protein, carbohydrates and fiber, must offer adequate amounts of fat (total and saturated) and sodium, according to the type of meal and the requirements of the assisted community, and must offer servings of varied fruits and vegetables.[6]

Throughout the nearly 40 years of existence of the WFP, numerous studies have been developed in order to assess the political aspects, the dietetic composition of the supplied meals, and its relationship to health and workers’ productivity.[7–10] However, comparative studies with prospectively collected data assessing the program by focusing on nutritional indicators of its clientele are not found in the literature. Therefore, the objective of this study, which was successfully accomplished, was to assess whether there was an association between the Brazilian Workers' Food Program and the nutritional status of workers in the transformation industry.

## Methods

We conducted an observational, cross-sectional survey based on probability samples, with prospectively collected data, comparative of workers from industries adherent and non-adherent to the WFP. This study was approved by the Institutional Review Board of the University Hospital Onofre Lopes with the number 776.772/2014. The survey was restricted to the transformation industry sector, because of its greater contribution, compared to other sectors, to the economy and the labor market of the State of Rio Grande do Norte, and to small (20 to 99 workers) and medium sized (100 to 499 workers) industries, where the administrations agreed upon the conduct of the study.

For the selection of workers within each group, we adopted a combined proportional stratified and multi-stage sampling plan. The stratification factor was the company size with two levels, small and medium size. A second stratification factor was added, consisting of the sector of industrial activity: alimentary, non-metallic minerals and textile, which are the three major sectors of industrial activity in the State. For each group, adherent and non-adherent to the WFP, and for each stratum, a two-stage random sample of workers was obtained. The first stage consisted of a simple random sample of companies within each sector of activity with size proportional to the total number of companies operating in that area. The second stage was a simple random sample of workers within each company selected in the first stage. Sampling frames were constructed for the two stages. For the first stage, a list of all companies operating in the State was obtained from the Federation of the Industries of the State of Rio Grande do Norte (FIERN); for the second stage, a list of all the refectory users at lunch hour was obtained from each company selected in the first stage. The randomization procedure adopted for the two stages consisted of assigning to each element in the list a random number generated with the runiform() command of Stata 11 Statistical Package (Stata Corporation, College Station, TX, USA) and selecting for the sample the elements with the lowest values until the required number of elements was selected. All workers selected in the second stage that gave their written informed consent were included in the survey. No worker declined or withdrew consent for study participation.

The primary study variable was the Body Mass Index (BMI), defined as the weight in kilograms divided by the square of body height in meters. The survey was sized to detect, with 80% power, a difference in BMI between groups adherent and non-adherent to the WFP of 1.6 kg/m2, assuming a standard deviation of the BMI of 6 kg/m2. This difference of 1.6 kg/m2 between the two groups corresponds to an effect size of 0.27, which is considered a small effect. For logistic reasons, we decided that 40 workers should be selected in each company, which led to a total sample size of 1040 subjects in 26 companies, assuming an intraclass correlation coefficient of the BMI of 0.03. This sample size includes 80 additional observations to compensate for covariates used in the statistical models. Therefore, the target sample size was established as 13 companies in each group, with 40 workers from each company for a total of 520 workers per group.

For the calculation of the sample size the following formula was used [11]:

$$n=\frac{2{\left({\text{z}}_{\alpha /2}+{\text{z}}_{\beta }\right)}^{2}\sigma^{2}\left[1+\left(m-1\right)\rho \right]}{\delta^{2}}$$

where *z*_*α*_/2 and *z*_*β*_ are the standardized deviates corresponding, respectively, to the 97.5th and the 20th percentile of the normal distribution (1.96 and 0.84), *σ*^2^ is the variance of BMI (assumed 36), *m* is the cluster size (defined as 40 workers), *ρ* is the intraclass correlation of BMI (assumed 0.03 [12]) and *δ* is the minimum difference between groups in mean BMI (defined as 1.6 kg/m2).

Before the study, the survey team was trained in the measurement of the primary study variables weight, height and waist circumference, according to the guidelines of the Alimentary and Nutritional Surveillance System (SISVAN) [13]. These variables were measured with a digital scale (Inner Scan, Tanita Corp., Tokyo), a body height meter (Sanny, São Bernardo do Campo, SP, Brazil) and a tape meter (Macrolife Ltda, Pato Branco, PR, Brazil), respectively. Nutritional classes according to BMI were defined as proposed by the World Health Organization [14]. In addition to anthropometric measurements (body weight, height and waist circumference), study data included age, sex, education, income, civil status, number of children, whether the worker attended some form of in-house formal professional training, blood pressure, level of physical activity [15] and comorbidities. Nutritional information on the composition of workers’ lunch was obtained with the 24-hour dietary recall method, using food models specifically developed for this study. Verbal information regarding food consumption during the lunch break in the previous day, was obtained from each worker by academic dietitians and by supervised graduate students previously trained in alimentary interviews. All the meal components were considered, including appetizers, main course, side dish, dessert and beverages, and were quantified according to previously established criteria (direct weighting, photographic records or food package labels) as portions sizes, following a model adapted from the ISA capital questionnaire [16]. Food consumption at other meals throughout the day was not considered, since the WFP is concerned only with food offered at lunch time. For nutritional analysis, the official 2011 Brazilian Table of Food Composition[17] was used, complemented when necessary by other food composition tables[18–20], and amounts were calculated for total energy, macronutrients (protein, carbohydrate, lipids and saturated fat), sodium and fiber, which are the nutrients described in Ordinance no. 66 [6] that defines the nutritional parameters for the WFP.

Statistical data analysis was done with the svy class of commands of Stata 11 Statistical Package (Stata Corporation, College Station, TX, USA). Population estimates of the characteristics of workers and of the study variables were obtained for the State of Rio Grande do Norte by using sampling weights consisting of the inverse of the probability of a worker being selected to the sample. The weights were computed from official data of the number of workers in each company and considered the stratification and the multi-stage design. Standard error estimates were corrected for clustering of workers within each company, based on the intraclass correlation coefficient, and for sampling without replacement using a finite population factor computed from the total number of industries in the State, according to the sector of economic activity, and from the total number of workers in each company selected to the study.

In order to identify characteristics of workers that are distributed differently between companies adherent and non-adherent to the WFP, we used nested mixed effects linear regression [21] for interval dependent variables and nested mixed effects logistic regression [22] for binary variables. Those variables with evidence (p<0.05) of different distribution between study groups were entered as covariates in the primary study analysis. The primary study variable was BMI. The secondary study variables were waist circumference and nutritional diagnosis. For comparing the former two variables between study groups we used nested mixed effects linear regression. The fixed effects were the study group (WFP or non-WFP), company size (small or medium), worker’s sex and age, and the covariates identified in the previous analysis. The random factors were the sector of activity (alimentary, mining or textile) and company nested within sector of activity. Company size and sector of activity were stratification variables, and age and sex are variables known to be associated with the dependent variables. Regression coefficients were estimated with the method of restricted maximum likelihood. Nutritional diagnoses were compared between groups using ordinal logistic regression with covariates company size, sector of activity, sex, age, education, income and training, with robust standard errors computed with the Huber-White sandwich estimator [23]. Calories and nutrients consumed by workers were compared between groups with the same model of nested mixed effects linear regression as above, with adjustment by the same covariates. Results are presented as point estimates and 95% confidence intervals (CI). Statistical significance was considered for two-sided p-values < 0.05.

## Results

Between September and December 2014, we evaluated 1069 workers in 26 companies: 541 workers from 13 companies adherent to the WFP and 528 workers from 13 companies non-adherent to the WFP. S1 File contains the complete dataset for this study. The distribution of the companies and study participants within each group is shown in Table 1.

**Table 1:** Characteristics of the surveyed companies in the two study groups according to the sector of economic activity and the company size.

Characteristics of surveyed companies	non-WFP		WFP	
	nb. of companies	nb. of participants	nb. of companies	nb. of participants
Sector				
Alimentary	5	181	5	218
Mining	3	123	2	77
Textile	5	224	6	246
Size				
Small	7	260	6	240
Medium	6	268	7	301

Table 2 shows the population estimates of the workers’ characteristics in the two study groups, as well as the results of the comparison of the distribution of these characteristics between groups. Workers of WFP-adherent companies are more likely to have greater school education (69% vs. 37% with middle school level education or greater, p<0.001), a larger income (45% vs. 30% with income above minimum wage, p = 0.04) and more frequent attendance of in-house training programs (28% vs. 16%, p<0.001).

**Table 2:** Workers’ bio-demographic and socioeconomic characteristics in companies non-adherent and adherent to the WFP in the State of Rio Grande do Norte, Brazil, 2014.

Characteristic	non-WFP			WFP			p
	(n = 528)			(n = 541)			
	mean/%	95% CI		mean/%	95% CI		
Age (mean)	34.5	32.4	36.7	34.5	33.2	35.8	0.77**
Male sex %	64.70	48.5	80.9	54.5	43.9	65.1	0.41*
Living alone %	44.1	37.6	50.5	46.1	39.5	52.6	0.75*
Children (mean)	1.5	1.2	1.9	1.3	1.2	1.5	0.30**
Education>middle school %	36.9	27.1	46.6	68.7	60.7	76.8	<0.001*
Income > minimum wage %	29.6	20.1	39.1	45.2	36.0	54.4	0.04*
In-house training %	16.4	8.7	24.1	27.6	21.8	33.3	<0.001*

* Nested mixed effects logistic regression;

** Nested mixed effects linear regression. Fixed factors: WFP and company size; random factors: sector of economic activity and company nested within sector.

The mean values of the main study variables, BMI and waist circumference, as well as their distribution by nutritional classes defined according to the BMI, are shown in Table 3. After adjustment by age, sex, education, income and in-house training, workers in companies adherent to the WFP have a mean BMI which is 0.99 kg/m2 higher (95% confidence interval 0.37 to 1.61 kg/m2, p = 0.002) and a mean waist circumference that is 1.53 cm greater (95% confidence interval 0.07 to 2.99 cm, p = 0.04) than the corresponding values of workers in companies non-adherent to the WFP. The prevalence of workers with excessive weight (BMI ≥ 25 kg/m^2^) in companies adherent and non-adherent to the WFP is, respectively, 62.6% and 55.5% (p<0.001), the prevalence of workers with increased waist circumference is, respectively, 23.3% and 20.6% and the prevalence of substantially increased waist circumference is, respectively, 25.9% and 19.3%. These differences are not explained by a difference in the level of physical activity, which was not statistically different between groups (45.3% and 47.5% with moderate or intense physical activity in WFP and non-WFP respectively, p = 0.79).

**Table 3:** Frequency distribution of workers by nutritional diagnosis in companies of the transformation industry non-adherent and adherent to the WFP in the State of Rio Grande do Norte, Brazil.

Variables	non-WFP			WFP			p
	(n = 528)			(n = 541)			
	point estimate	95% CI		point estimate	95% CI		
BMI kg/m^2^ (m, sd)	26.0	25.5	26.6	27.0	26.5	27.4	0.002*
Adult BMI classification. [14] (n,%)							<0.001**
underweight (<18.5)	1.9	0.7	3.1	0.6	0.0	1.4	
normal weight (18.5–24.9)	42.6	36.2	49.0	36.6	32.7	41.0	
excessive weight (≥25.0)	55.5	49.1	61.9	62.6	58.4	66.7	
overweight (25.0–29.9)	40.3	35.3	45.2	41.0	36.5	45.5	
obesity I (30.0–34.9)	12.3	8.8	15.8	15.7	11.8	19.6	
obesity II-III (≥35.0)	3.0	0.8	5.2	5.9	3.0	8.8	
Waist circumference cm (m,sd)	86.5	85.3	87.7	87.9	86.9	88.9	0.04*
Waist circ. classification ^15^ (n,%)							0.02**
normal	60.1	50.3	70.0	50.9	44.6	57.2	
increased	20.6	15.9	25.3	23.3	18.0	28.5	
substantially increased	19.3	12.6	25.9	25.9	21.8	29.9	

* Nested mixed effects linear regression; Fixed factors: WFP and company size; random factors: sector of economic activity and company nested within sector.

** Ordered logistic regression. All analyses adjusted by age, sex, education, income and in-house training.

The analysis of the consumption of energy and of nutrients at lunch (Table 4) showed, for the WFP group, a decrease intake of sodium (1.81g vs. 1.99g, p = 0.01) and of saturated fat (6.7g vs. 9.8g, p = 0.01), compared to the non-WFP group. These differences could be due to the greater prevalence of overweight observed in the WFP population, that could be associated with a higher prevalence of hypertension and consequent sodium restriction. However, we have found no statistical differences between groups, neither in the mean levels of systolic (p = 0.54) and diastolic (p = 0.39) pressure, nor in the proportion of workers either high blood pressure or being medicated with anti-hypertensives for a previous diagnosis of hypertension (p = 0.35).

**Table 4:** Calories and nutrients consumed at lunch by workers of companies the transformation industry adherent to the WFP compared to workers in non-adherent companies in the State of Rio Grande do Norte, Brazil.

Variables	non-WFP			WFP			Difference			p
	(n = 528)			(n = 541)						
	mean	95% CI		mean	95% CI		mean	95% CI		
Energy (Kcal)	750	667	832	743	675	811	-33.8	-117.8	50.2	0.43
Protein (Kcal)	192	174	210	186	170	203	-13.6	-34.5	7.3	0.20
Lipids (Kcal)	214	181	246	211	180	242	-18.5	-57.6	20.7	0.36
Carbohydrates (Kcal)	346	308	385	351	318	383	0.4	-37.0	37.9	0.98
Fiber (g)	15.7	13.9	17.5	15.0	12.8	17.2	-0.55	-2.70	1.59	0.61
Sodium (g)	1.99	1.77	2.22	1.81	1.50	2.11	-0.30	-0.53	-0.07	0.01
Saturated fat (g)	9.8	4.4	15.2	6.7	6.0	7.3	-1.34	-2.38	-0.30	0.01

* Nested mixed effects linear regression; Fixed factors: WFP and company size; random factors: sector of economic activity and company nested within sector; adjustment by age, sex, education, income and in-house training.

## Discussion

The results of our survey showed that the workers' nutritional status, regardless of adherence or not to the WFP, is in the pre-obesity range, thus following a trend of populations in the world at large.[24,25] Among workers of the WFP group, however, there was a higher BMI and waist circumference compared to workers of the non-WFP group. Since the study controlled other variables influencing body weight, this finding strongly suggests an effect of the WFP on the nutritional status of workers. We have found no evidence of differences between WFP and non-WFP groups in calorie and macronutrient intake at lunch time, as would be expected given the difference in BMI. We believe that increased BMI in the WFP group may be more related to a regular food supply and to different choices of food groups, than to caloric intake. In order to verify the latter hypothesis, we are now conducting a qualitative evaluation of food consumption and of food choices between WFP and non-WFP workers. Retrospective cohort studies that assessed food programs in workers of the Brazilian State of Bahia have also shown a statistically significant positive association between weight gain/overweight and being a worker at a company adherent to the WFP, when compared to a worker not benefited by any food program within the workplace.[7,8] However, the evidence produced by these studies was weak since they were based on secondary data, which were obtained retrospectively from the clinical charts of workers from convenience samples of companies in the industry sector. Therefore, previously to this study there have been no reports in the literature on the evaluation of the WFP through studies based on probability samples and prospectively collected data. To the best of our knowledge, this is the first time that such methodology has been applied in the assessment of the program.

Worldwide, public policies governing worker food programs are not recent. The first records of these programs date from the 1950’s in the United Kingdom, when restaurants and nearby companies agreed to supply adequate meals upon presentation of a ticket, for which the restaurant was subsequently reimbursed by the company. This occurred after the World War II, when the countries involved needed to recover in terms of their economies and human issues, making that initiative a social necessity. This became popularized, prompting the British government to concede total tax exemption in 1954.[26]

From then on, other European countries and those from other continents also implemented in the mid-1960’s the ticket system to subsidize workers’ meals, by offering fiscal deductions for the entire or partial value invested by participating companies. These included Hungary, Romania, France, Sweden, India, Lebanon, China and Brazil. France’s policies developed quickly and continue to play an important role in this segment of society, while Hungary, the country with the widest coverage, reached 80% of its workers due to strong union participation.[27]

The trend towards overweight observed in our study as well as by others [7,8,28] is associated to the predisposition to, or worsening of, several chronic non-communicable diseases, as the World Health Organization (WHO) considers it a major risk factor.[29] This reinforces the need for food policies whose actions include the provision of meals to the target clientele, to have predefined guidelines of nutritional coverage and to establish control mechanisms of such coverage, because it is known that the best strategy for preventing and tackling overweight and obesity is the dual approach based on a balanced diet and regular physical activity.[30]

The results of the analysis of cardiovascular risk of workers demonstrated greater prevalence of workers with increased risk and substantially increased risk, according to the classification of the WHO [14] in the WFP group. This result is related to the observation of higher BMI among WFP workers, since the risk was measured by waist circumference, i.e. a body measure subject to change with weight gain. This finding is somewhat worrisome because, although the prospect of the WFP is the weight gain of workers as a reflection of the improvement in the nutritional status, this gain should be related to an increase in lean mass, rather than fat. The results of this study seem to reveal what authors in the area of policy evaluation refer to as a perverse effect, which is when, while the results expected by the action of the policy have been achieved, through the transformation of a given situation the policy leads to undesired results and to a modification of the previously faced problem.

It is important to point out that when the WFP was established, in 1976, in the epidemiological profile of Brazil at that time workers were a population group vulnerable to deficiency diseases. It was therefore urgent that the government turned its attention to that group. The dominant logic was that improving the nutritional status of workers would increase productivity. With the passage of time, the program goals evolved to encompass the commitment to the health and the quality of life of workers.[31]

Political, economic and social changes in Brazil reflect, to some extent, the current epidemiological profile. These changes are also the result of many changes that occurred in Brazil in the last two or three decades. Firstly, due to the rural exodus, which contributed to the urbanization of society, secondly, because the patterns of work and leisure, food and nutrition, and health and disease have become close to those found in developed countries.[32]

Moreover, the regular and sustained access to food in adequate quantities is part of the concept of food security and that is what the Workers’ Food Program offers daily to its clientele. It is to be expected that this regularity promotes weight gain. On the other hand, attention to the qualitative aspects of the food that is being supplied, with attention being paid to the energy balance and the harmonic distribution of the nutrients, in addition to the monitoring of the nutritional status of workers, can encourage that this weight gain takes place in a healthy manner, without compromising the workers’ health. Finally, since the WFP is a policy carried out within corporations, it is an excellent means of disseminating information on healthy eating habits. Studies have demonstrated the relevance of implementing strategies that enable the creation of environments that promote a healthy diet and physical activity as mechanisms to control obesity.[33]

Workers in WFP-adherent companies are more likely to ingest lesser amounts of saturated fat and salt, a positive finding since a high intake of these food components may increase the risk of obesity and promote the development of cardiovascular and other chronic non-transmissible diseases.

There are some limitations of this study. This was an observational study, not an experiment with random allocation of subjects to each group. Therefore, although care was taken to control for subject characteristics associated to nutritional status and to several confounding factors, a causal relationship may not be established. There could be systematic differences between companies adhering or not to the WFP that might influence the observed results. Company size and sector of activity could be influencing factors, but these were controlled for by stratification. In addition, the only two criteria for adhesion to the WFP are payment of corporate tax according to the actual profit method, as opposed to the deemed profit method, and to have workers’ salaries below 5 minimum wages. In all our analyses, we adjusted for worker’s income and we it is reasonable to consider that the tax payment method adopted by the companies has no influence on the nutritional status of workers. There may be differences between groups in the level of automation of the companies or in other characteristics of the work environment that might lead to a different number of expended calories. However, the analysis of level of physical activity, which included an assessment of the physical activity in the workplace, did not show statistically significant differences between groups. There is no information on the workers’ BMI and waist circumference before entering the Program, which raises the possibility, although unlikely, that there exists a selection bias whereby subjects with greater BMI are more prone to select a company beneficiary of the WFP. In order to assess an order factor between the WFP and BMI and WC increase, we are now conducting a long term evaluation at 4 years of the participants in this study. The study was conducted in a single State in Brazil, which may limit the generalization of the results, although studies with different methodologies conducted in other States have produced results that are consistent with ours. An unexpected finding was that higher rates of overweight and obesity were seen among WFP group workers, which differs from most studies that point to a lower prevalence of overweight in populations with higher levels of income and education. This, however, does not affect the results obtained in this study, since the analyses were adjusted for these variables.

## Conclusion

In conclusion, the results obtained in the present study, which assessed anthropometric measures of the nutritional status of workers benefited by the Workers’ Food Program, revealed statistically significant differences between mean BMI and waist circumference of the non-WFP and WFP group. One positive finding was the lower level of consumption of sodium and saturated fat among WFP workers, indicating a favorable association of the WFP with the nutritional intake of the population served by the program. Therefore, from a public health perspective, we believe that these results provide an important contribution by showing how an initiative by a central Government can contribute effectively to improving the nutritional status of a vulnerable population group in nutritional terms, without the need for the allocation, mobilization and direct application of national funds. In this sense, these results might encourage other countries, with similar population groups in vulnerable situations, to find in this model of public policy one way to combat the hunger and malnutrition, following the example of Brazil.

On the other hand, our study also demonstrated, for the first time, a higher cardiovascular risk among WFP workers, as reflected by a larger waist circumference. Consequently, our results make a strong point toward the need of periodically revisiting the WFP and redefining its goals according to the changing nutritional status of the target population of the program, in order to help workers maintain a body weight within healthy limits.

## Supporting information

S1 File Complete dataset. (XLSX)
